# Mastering the art of sectioning: a comprehensive guide to slide-microtome technology and histological applications

**DOI:** 10.3389/fvets.2025.1635706

**Published:** 2025-08-25

**Authors:** Yasmin M. Abd El-Aziz, Mohammed S. Sobh, Hebatallah M. Saad, Menna H. E. Morsy, Ehab El-Haroun, Kasim Sakran Abass, Zulhisyam Abdul Kari, Roshmon Thomas Mathew, Moaheda E. H. Eissa, El-Sayed Hemdan Eissa

**Affiliations:** ^1^Department of Zoology, Faculty of Science, Port Said University, Port Said, Egypt; ^2^Pathology Department, Faculty of Veterinary Medicine, Zagazig University, Zagazig, Egypt; ^3^Pathology Department, Faculty of Veterinary Medicine, Matrouh University, Marsa Matruh, Egypt; ^4^Zoology Department, Faculty of Science, Arish University, Arish, Egypt; ^5^Department of Integrative Agriculture, College of Agriculture and Veterinary Medicine, United Arab Emirates University, Abu Dhabi, United Arab Emirates; ^6^Department of Physiology, Biochemistry, and Pharmacology, College of Veterinary Medicine, University of Kirkuk, Kirkuk, Iraq; ^7^Department of Agricultural Sciences, Faculty of Agro-Based Industry, Universiti Malaysia Kelantan, Jeli Campus, Jeli, Malaysia; ^8^Fish Resources Research Center, King Faisal University, Al Ahsa, Saudi Arabia; ^9^Biotechnology Department, Fish Farming and Technology Institute, Suez Canal University, Ismailia, Egypt; ^10^Fish Research Centre, Faculty of Environmental Agricultural Sciences, Arish University, El Arish, Egypt

**Keywords:** blades, artifacts, microtome types, sectioning techniques, slides, tissue preparation

## Abstract

The well-known technique of microtomy, which is an essential cutting tool, was first developed for light and transmission electron microscope uses, but it is currently also utilized to prepare specimens for atomic force microscopy (AFM), ion microscopy using a focused ion beam (FIB), and scanning electron microscopy (SEM). Ultramicrotomy can only be used on soft substances and metals that are sufficiently ductile to be cut with a diamond knife. Before being sliced by a microtome, many soft materials must first go through numerous preparatory processes. The choice of microtome type and blade material depends on the specimen being cut and the desired thickness of the sections. The scope of this review is to provide an overview of the main types of microtomes and a comparison between various common types of microtomes, recognizing and classifying frequent difficulties in tissue sample preparation with a particular emphasis on sectioning and staining problems. This review will put your focus on some problems, such as thick section borders, prolonged fixation or excess aldehyde concentration, low ethanol concentration or incomplete fixation, and sections not sticking to slides, especially during staining. In addition, explaining the artifacts during the processing of specimens using a microtome along with discussing the applications, calibration, maintenance of the microtome, as well as its troubleshooting, in addition to future trends, as well as challenges for this tool. Studying these issues will elevate key insights regarding the necessity of exact fixation, temperature control, accurate sectioning techniques, and gentle handling of tissues for the quality of histological samples used for microscopic studies.

## 1 Introduction

Biological specimens are sliced into extremely tiny pieces for microscopic inspection using a mechanical device called a microtome (1, 2). Tome means cut, and Micro means little. This can be accomplished most often by sectioning blocks of tissues fixed in paraffin wax, although there are different ways to expose the tissue to microtomy. A tool for cutting is held in a microtome, which is the fundamental tool used in microtomy. Researchers and medical personnel may make tiny cuts in biological specimens, which are necessary for microscopic examination with this advanced technology. Solving the mysteries of cellular composition and operation through the skill of sectioning can lead to significant advances in medical research (3).

Steel, glass, or diamond-cutting blades are used in microtomes, according to the specimen being processed and the required section width. For light microscopy, histological slices of plant or animal tissue are prepared using steel blades. Glass knives are used to cut extremely fine slices for electron microscopic examination and to cut sections for light microscopy (4). For either light or electron microscopy, hard objects like bone, teeth, and stiff plant matter are cut with industrial-grade diamond blades. Additionally, thin pieces are cut for electron microscopy using gem-quality diamond cutters (5).

### 1.1 Historical background of microtome development

Many early devices relied on manually controlled mechanisms and were somewhat basic. Electrically driven microtomes were created because of advancements in response to the growing need for precision, which improved sectioning effectiveness and precision. Gaining an appreciation of the capabilities of contemporary microtomes requires a comprehension of this history (6).

In the early stages of the rise of light microscopes, razor blades were used by hand to trim plant and animal sections. It was discovered that to study the architecture of the specimen that was being observed, it was crucial to produce clean, repeatable incisions that allowed light to pass through, around 100 μm in diameter. This made it possible to observe specimens through light microscopes in the transmission phase (7).

George Adams, Jr. produced one of the earliest tools for making these cuts in 1770, and Alexander Cummings improved it (8, 9). Sections were made from the leading edge of the specimen using a hand crankshaft, and the apparatus was manually controlled. The specimen was contained in a cylinder. Andrew Prichard created a table-based concept in 1835 that made it possible to isolate the vibration by attaching the tool to the table and keeping the user and knife apart (3). Wilhelm His, Sr., an anatomist, has been linked with creating the microtome (10). The invention is also credited by certain accounts to Jan Evangelista Purkyně, a Czech physiologist. Several sources claim that the Purkyne model was the first to be used in practice (11). The initial microtomes were only cutting devices, and the early development period is mostly unknown, which answers the mystery surrounding the microtome’s origins. The creation of extremely thin and frequently tiny specimens by microtomy, in conjunction with the specific labeling of significant parts of cells or molecules, made it possible to see tiny characteristics at the culmination of the 1800s (12, 13).

The equipment has made it possible for researchers to work with precision, producing parts that would be impossible to produce by hand. In particular, it has made it possible to get intact object segments during study (14).

### 1.2 Types of microtomes: a detailed overview

This can be summarized as follows, based on their design and operational methods:

#### 1.2.1 Rotary microtomes

**Figure 1:**
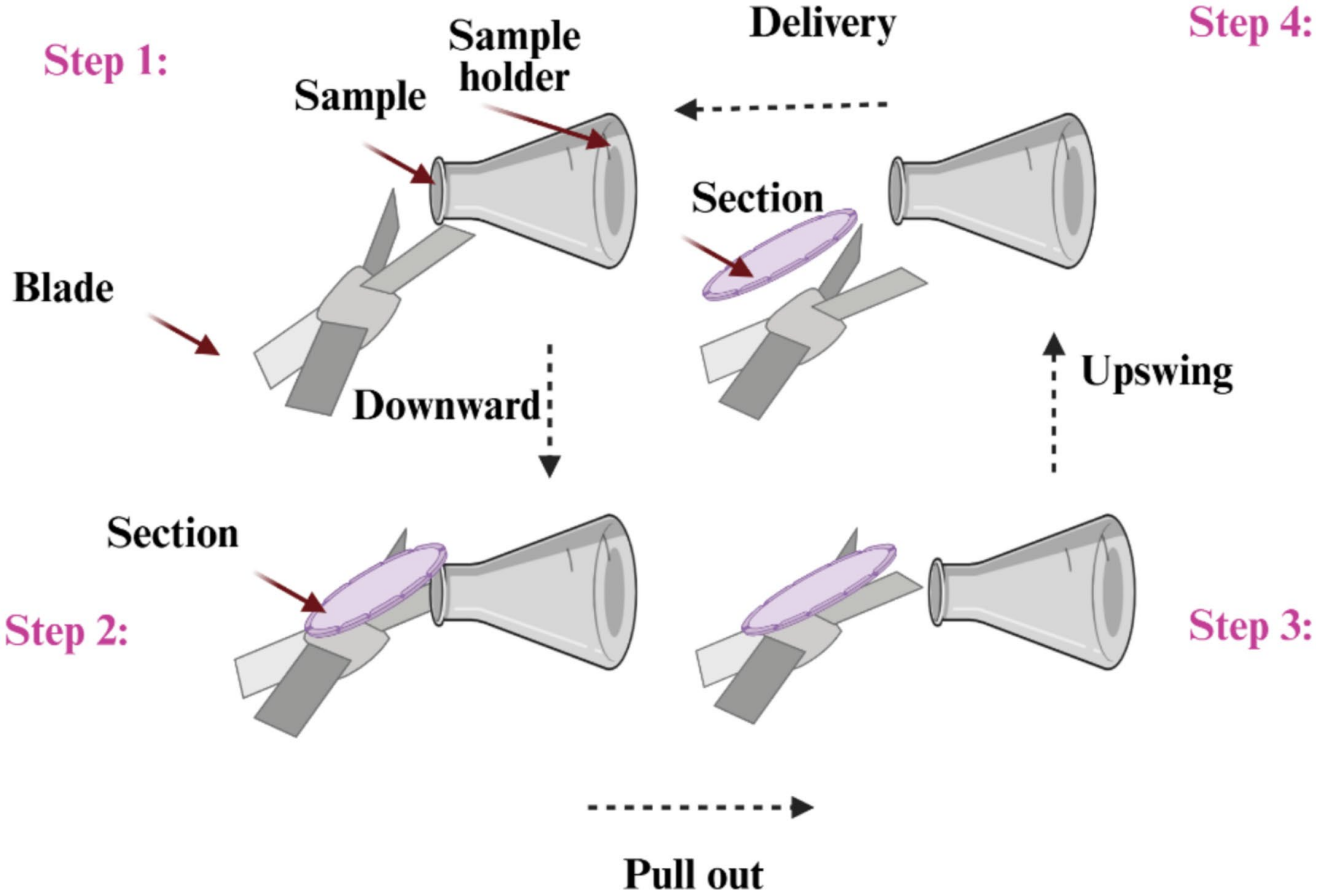
A specimen motion mechanism for cutting on a rotating microtome.

**Figure 2:**
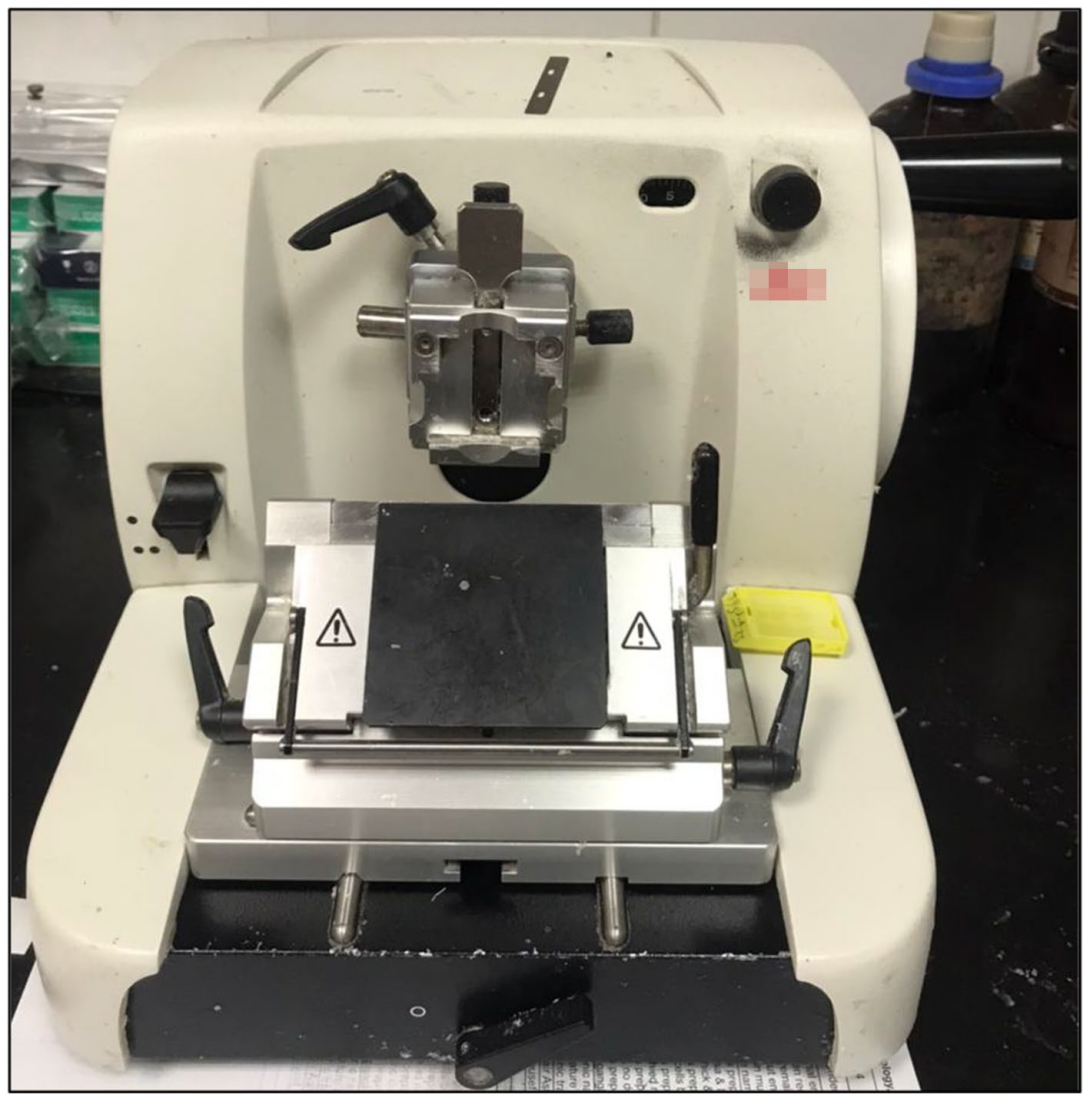
Representative photograph of a rotary microtome.

The most often used kind of microtomes are rotary ones (Figures 1, 2), which cut through paraffin blocks that are fixed on a holder using a revolving blade. They are preferred because they yield consistent paraffin sections, which makes them perfect for standard histology procedures. After that, the paraffin sections were put in a water bath at 37°C for smooth and straight spreading, then picked up using glass slides. This device is a typical microtome design. The real cutting is a part of the rotational motion in this device since it uses a staged rotary movement. The knife of a rotary microtome is usually set in a vertical position (15–18). The cut’s basic idea is described in the figure on the left. The fresh part stays on the knife after the specimen is sliced by the knife from position 1 to position 2 by the action of the specimen holder. The specimen holder advances by a comparable diameter as the section to be manufactured at the maximum rotating speed, enabling the creation of the subsequent section (19, 20).

Many microtomes use a hand-operated flywheel. The benefit of this is that straight cuts may be achieved since the flywheel’s comparatively enormous mass keeps the specimen from stopping while it is being cut. In more recent versions, the flywheel is frequently incorporated into the microtome’s case. A rotary microtome’s cut thickness typically ranges from 1 to 60 μm (21, 22). This type of microtome design may produce excellent “semi-thin” slices with an average thickness of as little as 0.5 μm for hard substances, such as specimens embedded into synthetic resin. The ability to cut tissue of different uniformity is another advantage of rotary microtomes. A water bath that has been heated to around 10°C below the paraffin wax’s melting point is necessary, along with the microtome and the microtome blades (23).

#### 1.2.2 Cryostat microtomes

**Figure 3:**
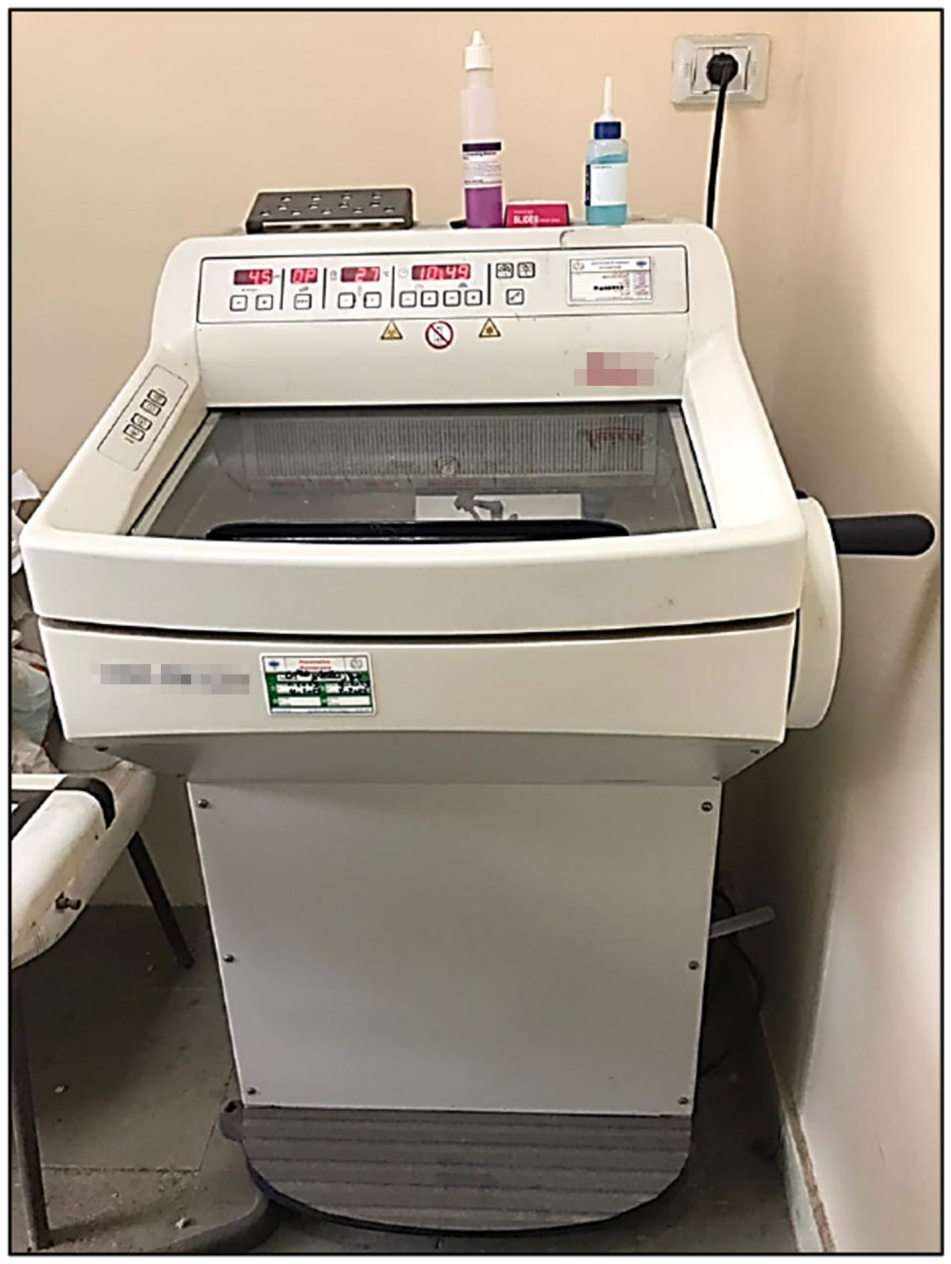
Representative photograph, cryostat.

Many rotary microtomes may be modified to operate in liquid-nitrogen chambers, a process known as “cryomicrotome setup” (Figure 3), to cut frozen materials. The creation of semi-thin specimens is made possible by the decreased temperature, which increases the specimen’s hardness through processes like a glass transition. To maximize the resulting specimen thickness, however, the temperature of the knife and the specimen must be regulated. In immunohistochemistry, this method is very helpful for maintaining the tissues’ native biochemical state (24).

The chuck is fastened to the cryostat’s specimen orientation tip for cutting when the tissue has sufficiently frozen. Using a microtome, tissue is typically sectioned at a measurement of 5 to 6 μm; specimens with a lot of fat may require thicker sections (e.g., 6–10 μm), whereas periocular tissue and tissue that needs immunostaining may require thinner sections (e.g., 4 μm). Minimize tissue facing, which is the process of using a microtome to remove tissue from the actual surgical margin before sectioning. Using a lot of tissue facing to get full sections that cover the whole margin may lead to false positives, requiring more layers and perhaps harming patients as well as adding time and expense (22, 25).

Before cryosectioning, the whole surgical margin can lie level on a single plane thanks to the glass slide embedding technique, which can help prevent excessive tissue facing. When cryosectioning, personnel may direct the frozen tissue slice onto the tip of the microtome blade using a camel hair paintbrush. The brush helps to avoid folds and smooth out wrinkles by gently holding the tissue as it proceeds to be cut. The tissue is then drawn to and adhered to the glass using a slide that has been warmed or slightly moistened. The specimen in question should be meticulously cleaned of any remaining embedding media (25).

#### 1.2.3 Sliding microtomes

**Figure 4:**
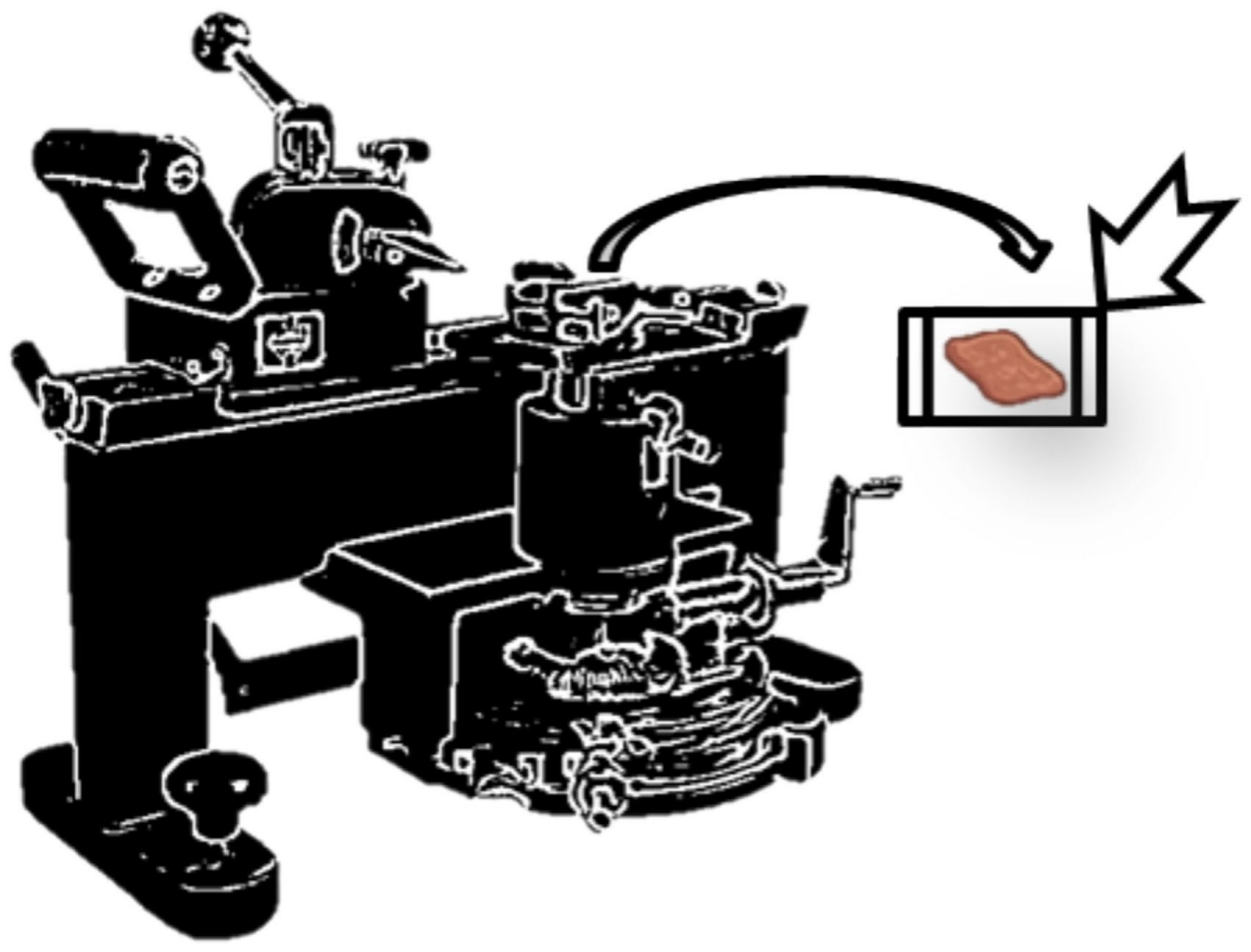
Representative photograph of a sliding microtome.

Sectioning is accomplished using sliding microtomes using sliding block technology. They are renowned for their durability in creating thick pieces and are frequently utilized for bigger specimens. The sliding microtome is a different kind of microtome in which a blade positioned vertically moves horizontally along a fixed block to cut a segment (Figure 4). Although it can also cut paraffin-embedded sections, the sliding microtome was created for celloidin-embedded sections. With its sturdy plastic design, fixed blade, and transparent plastic safety cover, this microtome allows students to securely and easily cut sections to 40–50 microns (26, 27).

#### 1.2.4 Ultramicrotomes

**Figure 5:**
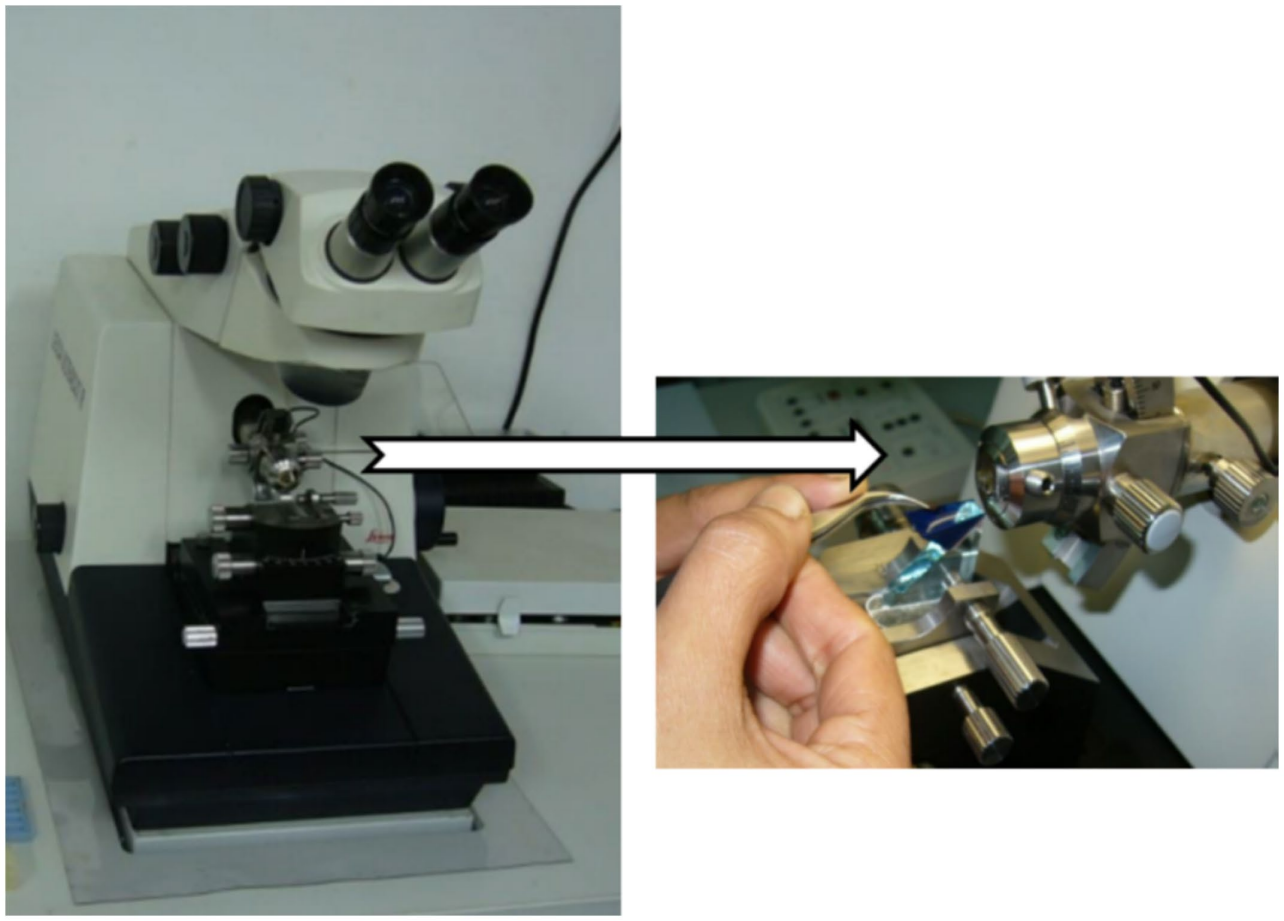
Representative photographs of an ultramicrotome.

The primary instrument used in ultramicrotomy is an ultramicrotome (Figure 5). Operating similarly to a rotating microtome but with exceedingly fine specifications on the mechanical structure, it enables the preparation of incredibly thin slices. Because of the meticulous mechanical design, the thickness may be precisely controlled by the mounting’s linear expansion during heating (24, 28). These incredibly fine slices are crucial for usage with serial block-face scan electron microscopes (SBFSEM) and transmission electron microscopes (TEM), and they are occasionally also crucial for light-optical imaging (29). For transmission electron microscopy, these cuts are normally 40–100 nm thick, while for serial block-face scan electron microscopes (SBFSEM), they are frequently 30–50 nm thick. To choose an area for the final collection of thin sections, portions that are up to 500 nm thick are also collected for specialist TEM purposes or light-microscopy surveying sections. Ultramicrotomes are utilized using glass and diamond blades, preferably. The slices are gently lifted onto grids appropriate for viewing TEM specimens after being floated on the surface of a liquid while being sliced. The very thin specimen thickness results in thin film interfering with colors of light reflections, which can be utilized for determining the overall thickness of the section (22, 30).

## 2 Comparison of microtome types

Here is a brief comparison of some differences between various types of microtomes (Table 1).

**Table 1:** Comparison between various common types of microtomes.

Aspects of comparison	Rotary microtome	Cryostat microtome	Sliding microtome	Ultramicrotome
Type of specimen	Fixed or fresh	Fixed or fresh	Fixed or fresh	Fixed or fresh
Specimen Size	0.5–60 μm	55 × 80 mm.	80 × 60 mm	50–100 nm
Embedding medium	Paraffin or epoxy resin	OCT, which consists of polyethylene glycol and polyvinyl alcohol	Paraffin or celloidin	Epoxy resins or acrylic resins
Preparation	A time-consuming, requires a lot of labor work process	A time-consuming, requires a lot of labor work process. It is necessary to implant specimens in a mold that is filled with an aqueous-based solution and frozen.	A time-consuming, requires a lot of labor work process	A time-consuming, requires a lot of labor work process
Drawbacks	More expensive, and dangerous as the blade is oriented upward position	Hard to use and needs specific precautions for specific specimens	More dangerous	Require specific expertise and a long process of preparation

## 3 Working principles of slide microtomes

An integrated progression system to advance the paraffin block until it makes a connection with the instrument used for cutting, such as a knife or blade, after a predefined distance. After the specimen passes through this cutting surface vertically, a tissue slice is created. To get the most out of slide microtomes, it is crucial to comprehend their underlying concepts. In the fundamentals of functioning, a blade moves over a stationary object. Section thickness may be changed using a precise control system, allowing researchers to customize their specimens to meet particular needs (31, 32).

### 3.1 Blade selection and maintenance

Choosing the right blade is essential to producing high-quality sections. Steel and tungsten carbide are among the materials used. The sectioning procedure is more efficient when the blade is kept sharp through ongoing upkeep. The microtome knife is essential to producing high-quality sections. A sharp razor blade was the initial attempt to provide a sufficient cutting surface, but they rapidly grew dull. Using newly shaped glass, Latta and Hartmann observed edges that were sufficiently fine in 1950 (33). Microtomy essentially starts and finishes with a clean, sharp cutting edge. Although the advent of consumable blades has facilitated the creation of high-quality, thin sections, they are sometimes insufficient for sectioning tougher tissues, particularly bone. Since these tissues present the microtomist with the biggest obstacle, the need to have a sharp knife has not decreased. The material used to make the knife, or the form of the knife-edge, can be used to categorize microtome knives.

#### 3.1.1 Blade types

Many blade types can be explained as follows.

##### 3.1.1.1 Steel blades

**Figure 6:**
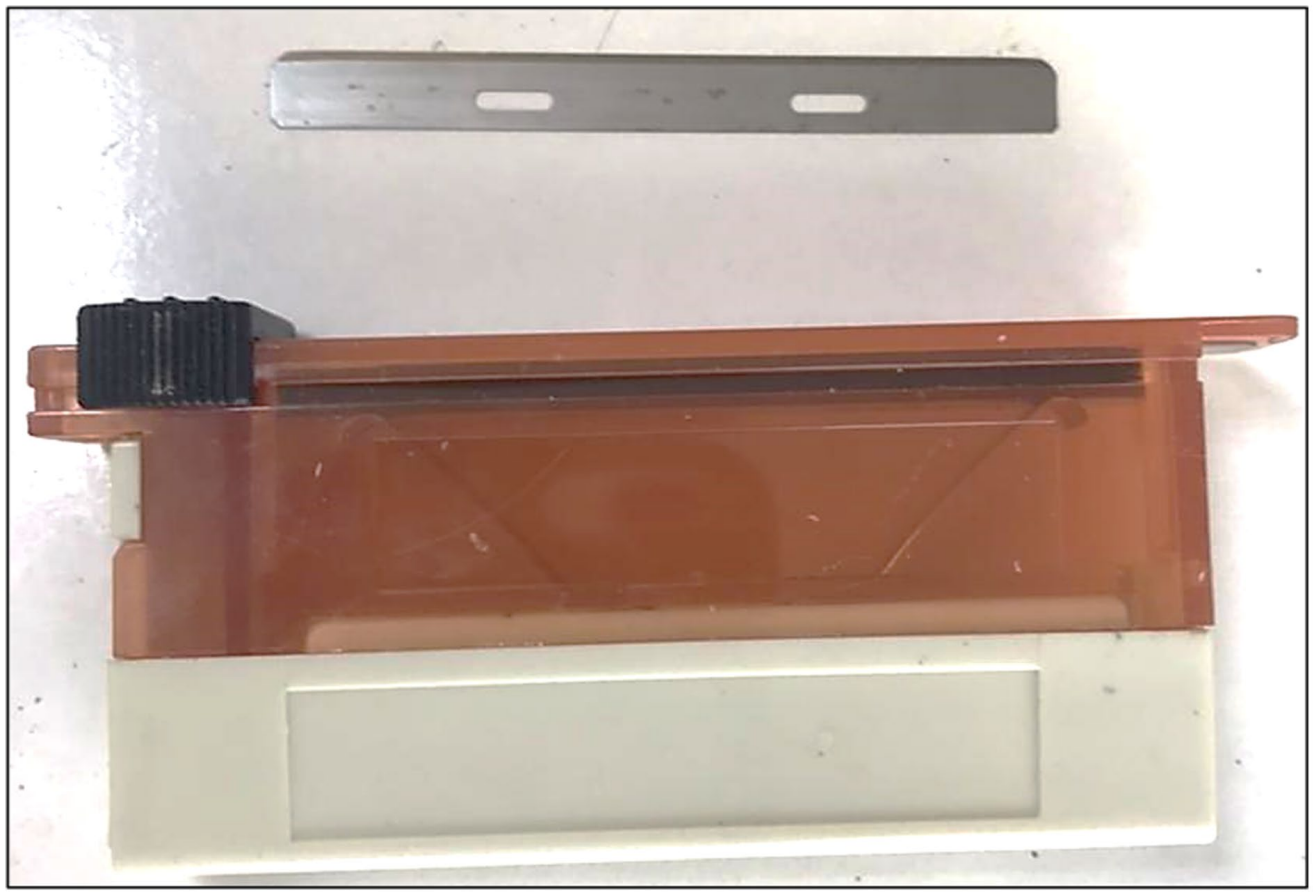
Steel microtome blades.

Steel microtome knives (Figure 6) are made from premium carbon or tool-grade steel that have been heat-treated to strengthen the edge (34). The steel should be devoid of impurities, have anti-corrosives, and be resistant to corrosion. It is the sharpened knives that are the best. Surface-hardened ones soon lose their cutting edge once the hardened region is removed by repeated re-sharpening (35). It is employed in broad plant or animal tissue histology sections.

##### 3.1.1.2 Glass blades

**Figure 7:**
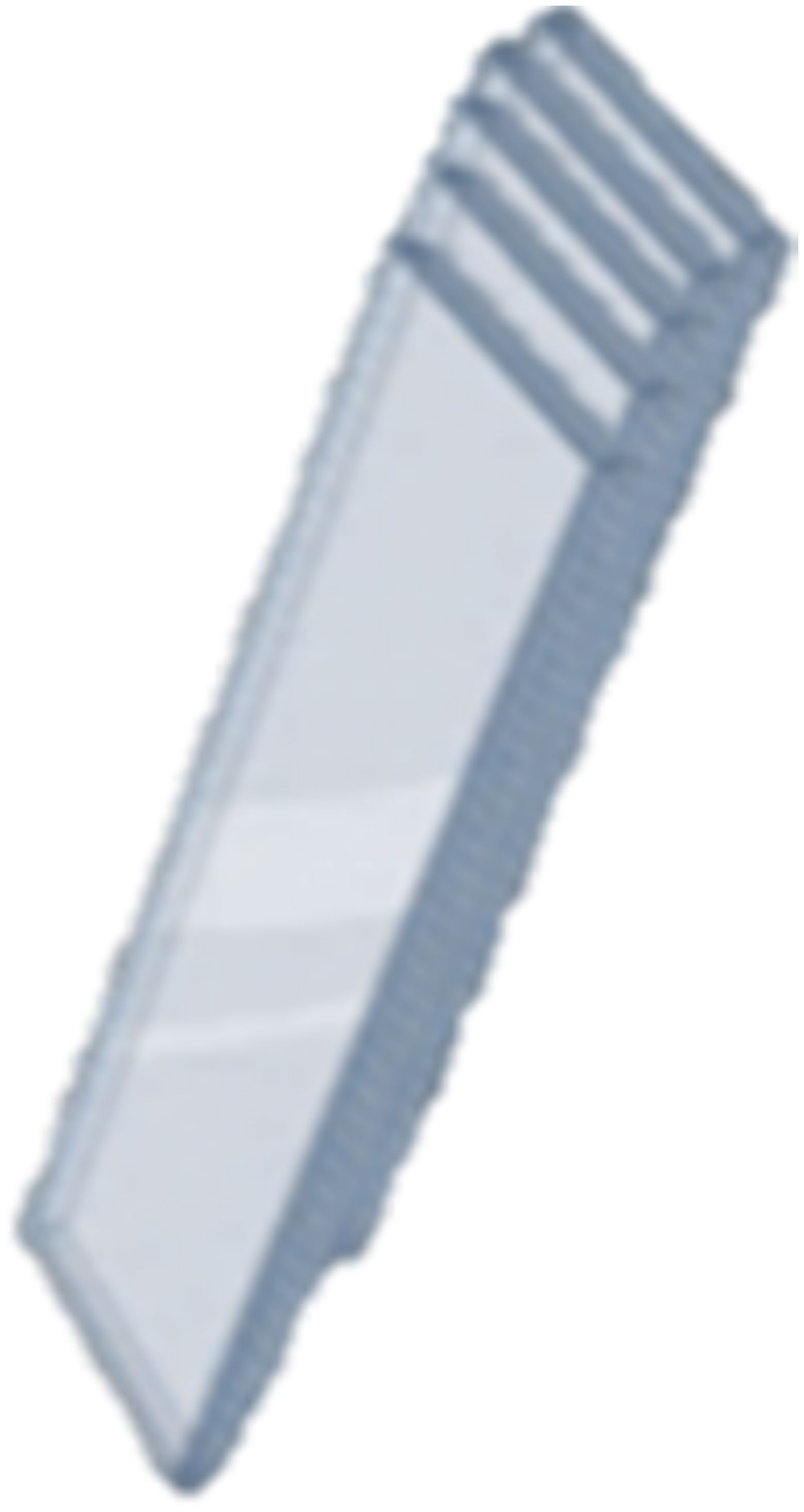
Glass blade.

Glass knives (also known as “Ralph knives,” which have edges of 25 or 38 mm) (Figure 7) used for traditional sectioning have their cutting blades parallel to one glass surface, whereas ultramicrotomy knives are positioned against or along the entire thickness of the glass. Rapid production of several “Ralph knife” profiles for slicing portions from various embedding media is possible. There are glass knife holders that allow “Ralph knives” to be utilized with a rotating microtome. Because glass blades are fragile despite their hardness, handling them requires caution. Due to oxidation impurities that remain in the hardened glass after manufacturing and changes in the “flow” or “strain” of the glass following fracture, these knives degrade with storage. Therefore, it is best to prepare knives just before using them (36). It is utilized for extremely thin slices, particularly in electron microscopy.

The shape of the edge that cuts may be used to categorize Ralph knives.

- High overview: cutting edge, 1.0 to 1.5 cm; ideal for cutting portions of soft embedding media, such as waxes.
- Intermediate characteristics: cutting edge height of 0.5 to 1.0 cm. It may be used to cut both hard and soft plastic portions.
- Low characteristics: the cutting edge is below half a centimeter in height. It is appropriate for cutting hard plastic parts.
- Front profile: insufficiently fastening the glass sheet at either end produces a sloping edge artifact that renders the knife unusable (35).

##### 3.1.1.3 Diamond blades

**Figure 8:**
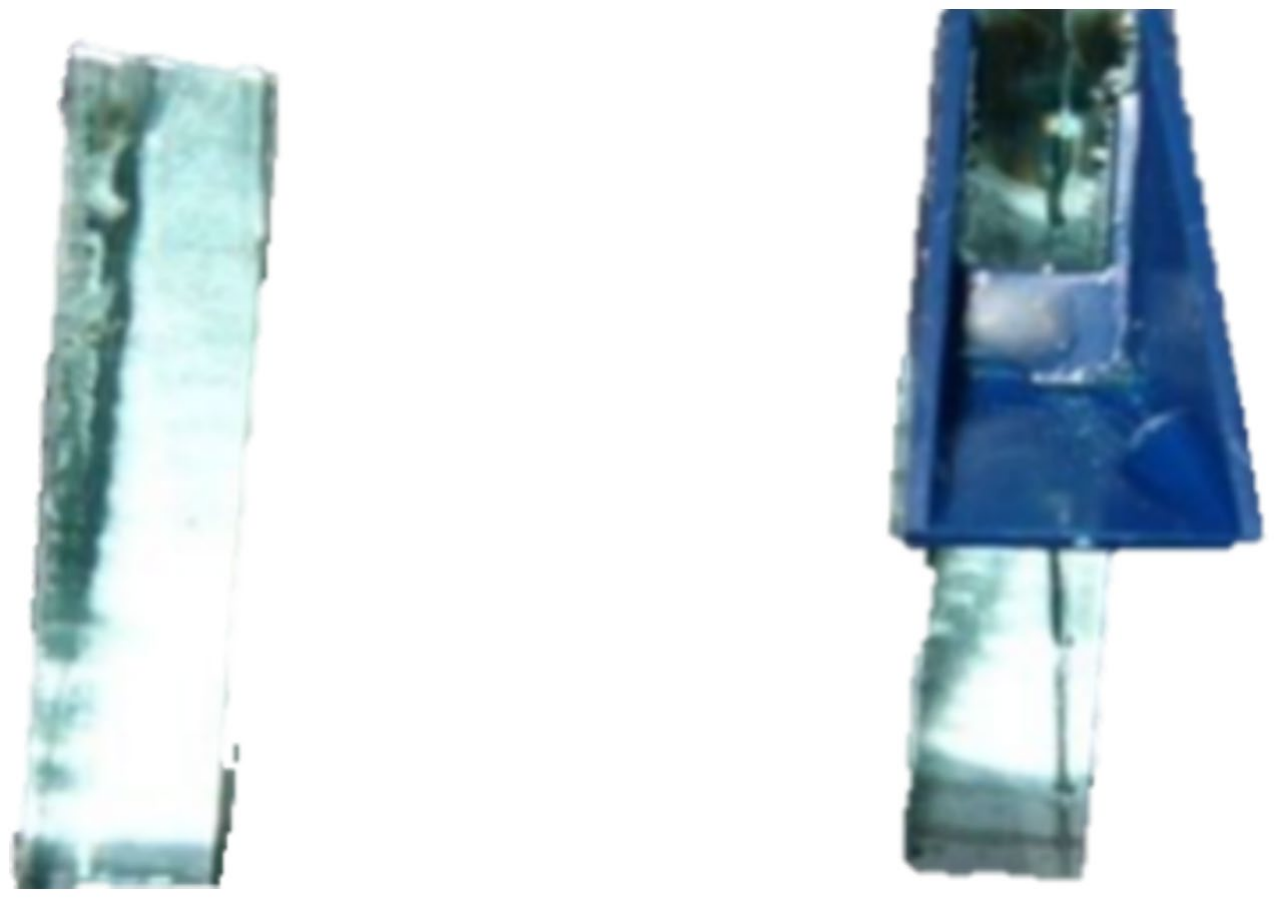
Diamond blade.

In 1955, Humberto Fernández-Morán developed the diamond knives (Figure 8), a highly sharp knife with a diamond-made edge (37). In scientific and medical settings where a very sharp and durable edge is necessary, diamond knives are employed. Depending on their quality and size, the knives can be quite costly to buy. As the edge dulls, they also need to be regularly sharpened. Diamond knives often cost several thousand dollars and are both delicate and costly. Diamond knives are often made from natural gemstones that are the purest possible, have uniform crystal structures, and are light yellow. The stones often lose half of their initial weight during the milling procedure. Wood’s metal, a soft metal shaft, is then used to affix the diamond blade, which is subsequently polished to an extremely sharp edge (38). The knife’s edge is incredibly sharp and flawless, making it easier to create ultrathin slices of uniform thickness for transmission electron microscopy (TEM) observations of specimens at high magnifications. Next, the shaft with the last edge is set in a metal “boat” or trough and cemented, often using epoxy resin. Atomic force microscopy also uses diamond blades to prepare specimens (39). This knife, which is utilized for high-precision electron microscopy procedures and for dissecting hard materials, comes in commercial and gem-quality categories (40).

## 4 Sectioning techniques and best practices

A good specimen preparation process depends on using the right sectioning procedures.

### 4.1 Specimen preparation

The steps for slide preparation differed according to the type of selected organs and the type of animal species. But, in general, slide preparation for soft tissues involves five sequential steps: (1) Fixation of trimmed tissues by using 10% neutral buffered formalin (NBF) for 24 h. (2) Dehydration of tissues in ascending graded levels of alcohol solutions (50% for 1 h, 70% for 1 h, 80% for 1 h, and 100% or absolute alcohol for 1 h). (3) Clearing of tissue specimens in xylene I for 1 h, xylene II for 1 h. (4) Embedding the tissue specimens in melted paraffin wax I for 1 h, and melted paraffin wax II for another 1 h. at 65°C. (5) Sectioning with a microtome produces thin tissue slices at 3–5 μm, which are transferred to glass slides. These tissue sections become ready for staining to make their components visible for microscopic examination (22, 41–43).

Specimens must be properly preserved, dehydrated, and embedded in a supportive media prior to sectioning. The quality of the sections generated can be greatly impacted by the embedding media selection. There are many different approaches to preparing specimens, ranging from straightforward and easy to intricate, laborious, and even irritating. Thankfully, some straightforward techniques work well for certain materials. For instance, the basic techniques can manage a lot of particle matter. Many of these simpler and more straightforward techniques are covered in this section and are broken down into three main subsections: optical microscopy, scanning electron microscopy (SEM), and transmission electron microscopy (TEM) preparations. It must be underlined that generally, materials may be seen quickly using a mix of direct preparation techniques and basic microscopy techniques, which frequently help to clarify the issue. This helps determine the appropriate course of action for a solution. There is frequently no one right way to do things, but if done early in the research, some methods could be able to save time (44–46).

#### 4.1.1 Optical setups

The stereo binocular microscope is the single most crucial preparatory tool in the microscopy lab. These tools are widely accessible and reasonably priced. Materials can be seen in either transmitted or reflected light, and the outcome frequently sheds information on the issue. The crucial initial step in selecting the region of a specimen to be investigated may include looking at even large portions. For transmitted light examination, transparent specimens, typically those with a thickness of less than 100 μm, can be put straight onto normal glass microscope slides using cover slips. This preparation could be enough for magnifications under 100 times. To minimize surface reflections, an appropriate mounting medium is often needed for greater magnification investigation (47, 48). While comparing refractive index oils can be employed with fibers and films to allow for the observation of internal frameworks, immersion oils with particular refractive indices or specialized mounting media, like Permount, can be used to create contrast among the material and the mountant in the case of particles. This method may be used to create fibers, particles, thin films, and membrane strips, and more for optical examination. Using a cavity slide to enable optical examination of a given fluid thickness is a direct way of fluid preparation. This method can be used to investigate crystal solutions. Solids or solutions can be put onto a slide with an outer cover or in a hollow slide (if necessary, in a dry or inert setting) (49, 50).

#### 4.1.2 Scanning electron microscope (SEM) setup

The ease of specimen preparation is a key benefit of using the SEM for surface investigations. In the most basic scenario, the substance to be examined is carefully selected from a larger specimen and is put on a specimen stub using double-sided adhesive tape. The item being examined is partially on the tape and partially on the stub because the tape only covers a portion of the specimen’s stub to retain contact with the stub for conducting. Silver or carbon solutions are examples of conductive paints that are used to adhere the item being studied to the specimen container. To make contact with the stub, these colors can also be applied over the tape or the specimen’s base. During handling, care should be taken to avoid mistreating the material. These straightforward preparation techniques work incredibly well for fibers, membranes, films, and even rather big plastic components. Since a conductive coat is typically needed for imaging, the preparation is not finished. A straightforward technique to reduce charging issues is to apply antistatic sprays like Duron. In Duron, Mather et al. (51) soaked and sprayed materials. This material’s dispersed droplets are visible at magnifications greater than 1,000x. Numerous specimen holders that are helpful for straightforward fiber and fabric preparations, where the specimen just must be connected to the holder. Different microscope stubs are provided by different SEM manufacturers and EM supplying laboratories, and machine shops may easily adjust (52).

#### 4.1.3 Transmission electron microscope (TEM) setup

**Figure 9:**
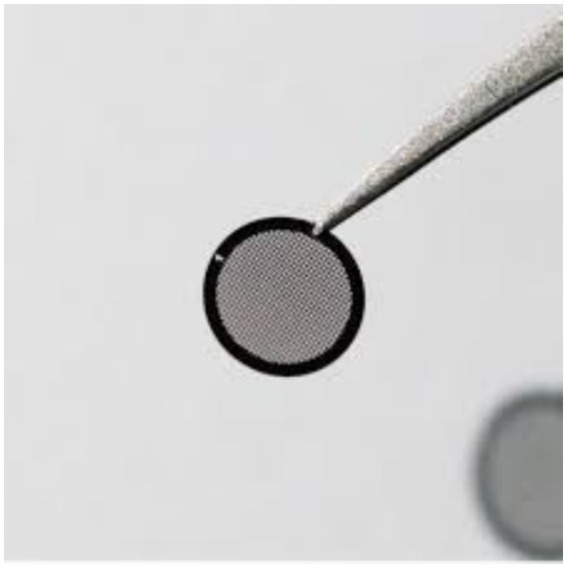
Grides (updated from Graticules Optics company for TEM grids).

Transmission electron microscopy specimens need to fit into a grid (Figure 9) or screen, which is a specimen support. This structure is a metal mesh screening that fits into the microscope’s specimen holder. It typically has a diameter of 2–3 mm. There are several mesh shapes and sizes available for grids. Commonly used sizes fall between 50 and 400 mesh, or 50 and 400 holes per inch. Although square grid meshes are commonly used, some materials may favor slotted displays, rectangles, hexagonal shapes, or single holes. Although copper makes up many grids, beryllium, gold, polymer, and nickel grids are utilized for a variety of purposes, including x-ray analysis and chemical resistance. Typically, microtomed sections that have polymer incorporated in a resin are supported directly on the grid. To keep them on the grid, very tiny or disintegrating parts would need support. On the TEM grid, particles, crystals, emulsions, and other fine substances are positioned on an electron-transparent support sheet. Below is a description of how to prepare these support films. TEM specimen preparation typically entails creating a thin layer of material that is less than 100 nm thick. The physical shape and composition of the polymer determine the techniques utilized for its preparation. Microtomy is typically used for thick or bulky specimens. Simpler techniques can yield a thin, scattered form of the substance in the case of liquids, powders, or particles (53, 54).

### 4.2 Reaching the ideal section thickness

For the planned analysis, the proper thickness must be determined. A variety of tissue types and study goals, standard slice thicknesses vary from 3 to 10 micrometers. However, upon changing the type of microscope thickness of the section may differ: For Widefield microscope, maximal thickness 20 μm, for Laser-scanning confocal = 100–200 μm, Spinning disk confocal microscope: 30–50 μm (55). In contrast, a focused ion beam is utilized to create a cross-section layer for the Scanning Electron Microscope (SEM), which measures membrane thickness for L < 20 μm. Additionally, the needed section thickness for transmission electron microscope (TEM) examinations usually falls between 50 nm (silver) and 100 nm (gold); however, this might vary in some situations (56).

### 4.3 Handling and mounting sections

Sections must be handled carefully after cutting to prevent ripping or deformation. To provide a smooth and level surface for microscopic inspection, slices must be precisely mounted on slides. Too thin a mounting medium creates air bubbles behind the coverslip, and as it evaporates, additional air is drawn under the edges. By removing air bubbles from beneath the slide and using mounting material that is sufficiently thick, this can be avoided (57). Additionally, it’s possible that dry mounting artifacts were seen. To fix this artifact, take off the coverslip, immerse it in xylene for a few minutes, and then remount it before the slide dries (58). Additionally, using mounting material excessively or too much may make the image look blurry. By employing a sufficient quantity of mounting material that is properly consistent, this phenomenon can be avoided (59).

### 4.4 Handling and orientation of specimens

Precise specimen orientation is essential for microscopy, although it is generally done with poor precision using macroscopic instruments. Because the specimen is embedded before imaging, it usually remains in a fixed orientation during the experiment and cannot be moved after it is placed in the microscope. In certain light-sheet microscopy (also known as Selective Plane Illumination Microscopy, or SPIM) (60) systems, a vertical rotating axis provides one degree of adaptive alignment of the specimen. Our non-contact specimen orientation strategy will be highly advantageous for high-throughput methods that do not yet provide a rotating axis for specimen orientation. By orienting all specimens similarly, the sensitivity and specificity of the screening process may be improved (61–63).

The acquisition of spectral data for electron microscopic analysis depends on an accurate understanding and control of the specimen’s orientation concerning the electron beam. A part of Fourier space is left unsampled, but most of it is used to gather data using tilting stages that can be +/− 70° around a single axis or +/− 65° with two points of independence (double-tilt or single-tilt and rotation). Because of the absence of symmetry, this is a significant barrier for many biological specimens, although it might not be a major one for the majority of crystals (64). In some instances, small portions of specimens were adhered to lens paper using cyanoacrylic glue right before fixation in glutaraldehyde. In addition to keeping the specimens together throughout tissue processing, the lens paper also acted as a “landmark” for the specimens, allowing them to be oriented in a certain way during implantation and subsequent sectioning (65).

## 5 Maintenance and care of microtomes

For microtomes to last and function well, frequent upkeep is essential. To guarantee safe handling and functioning, laboratory workers must obtain practical instruction from a certified supervisor; proper training is not negotiable. In addition to protecting the user’s hands and fingers, implementing safety procedures at every stage lowers the possibility of contaminants and harm to equipment. The following are important safety guidelines to assist in guaranteeing safe and efficient microtome operations while upholding the strictest laboratory safety regulations.

### 5.1 Take extreme care when handling blades

Because of their extreme sharpness, microtome blades pose a significant danger of harm. When installing and removing blades, always according to the manufacturer’s instructions. To prevent direct contact, use a dedicated blade holding tool if one is available (66).

### 5.2 Exercise caution around tungsten-carbide blades

In addition to being sharp, tungsten-carbide blades are hefty. They may easily sever shoes if dropped. When working with these blades, always place your feet in a stable and safe posture.

### 5.3 Properly keep blades

Blades should be kept in a closed container with stiff holding guides to avoid unintentional injury. This reduces the possibility of accidental contact and guarantees safe storage.

### 5.4 Steer clear of leaving blades on counters

Blades should never be left exposed to countertops. Serious cuts may arise by grabbing across an adjacent surface and unintentionally touching an exposed blade. Blades should always be stored safely while not in use.

### 5.5 Get the microtome ready

Before putting the blade in place, position the specimen correctly. This guarantees that the specimen is clamped firmly and reduces the possibility of unintentional cuts during setup.

### 5.6 Properly tighten the brake

Accidents are more likely to occur when a brake is unfastened. Before starting to cut, verify that the brake is firmly in place to avoid drawing the hand into the blade (67).

#### 5.6.1 Clearing protocols

Maintaining a regular clearing schedule helps avoid contamination and guarantees peak performance. Blade cleaning, part replacement, and maintaining components that move are all included in this. Start by donning your safety gear. Remove the knife gently with forceps. Immediately dispose of discarded knives in a sharp container. If you intend to use the knife again, soak it in a bottle of disinfectant, making sure the knife has full time to contact with the solution. Use alcohol to wash the experiment’s head and levers, clear the trash tray, clean the instrument’s blade holder of debris, and avoid using too much alcohol while using a cryostat. Check the temperature setting (22, 68, 69).

#### 5.6.2 Calibration and performance checks

For precision, microtome settings must be regularly calibrated and aligned. Potential problems can be found before they have an impact on section quality by conducting routine performance inspections. Microtome alignment may be done in two ways: either with blank blocks or using a microtome aligning tool. It is inserted into the clamp for the cassette. In the middle is a bubble. The microtome is in alignment when its bulge is in the middle of the black circles. The microtome has knobs for adjusting the cassette clamp. Before adjusting the alignment knobs, the microtome’s lever needs to be relaxed. The x-axis is adjusted by one knob, while the y-axis is adjusted by the other. The lever must be locked into position after the microtome is positioned. For precise instructions on how to adjust the knob for the cassette clamp, see the equipment handbook for the particular type of microtome in your laboratory. The use of blank blocks is another technique for aligning microtomes with one another. For every microtome you want to align in the lab, you should prepare an empty block, an object with paraffin only put into the mold, and a cassette on top. Verify that the unfilled blocks are flat. A uniform slice of paraffin should be cut if you insert a blank block into it. When you begin aligning misaligned microtomes, you may identify where the misaligned microtome is by observing the block’s cutting motion. The block’s direction sides may be sliced excessively; in this case, the x-axis must be modified (22, 70).

## 6 Common issues and troubleshooting in sectioning

Sectioning may be difficult in various ways, and troubleshooting can be made easier by knowing the usual problems, and these issues can be summarized as follows (20, 58, 71).

### 6.1 Cutting issues

#### 6.1.1 Make an angle cut

Cuttings for sectioning must be made perpendicular to the root’s longitudinal axis. This may appear simple, but it is far more challenging to execute and maintain throughout segmenting a whole segment. Until a cut is done to resurface the perpendicular surface, all parts that follow will be at an angle after the initial angle cut.

The following characteristics can be used to identify angled cuts:

A portion of the section or cells inside it appears to be “smeared” because of the oval shape of the cells, which allows one to concentrate across many cell layers in one location.

#### 6.1.2 Cut too thinly

If a segment is not entirely spherical or has a missing portion, this issue can be recognized.

#### 6.1.3 Cut too thickly

If the section’s borders seem unusually thick or if you can see through many cell layers throughout the whole section, you can identify this issue.

#### 6.1.4 The dull razor blade

Because of the blade’s cutting resistance, the researcher can unknowingly apply more pressure to the section or employ a sawing motion. As a result, portions that are typically oval in shape look “smeared” or deformed.

#### 6.1.5 Half-cut sections

The issue is that the water drop has too many pieces, and convection is pulling them back toward the blade, so they may be chopped in two. These issues may be fixed with careful adjustments and routine blade repair.

### 6.2 Issues associated with tissue

#### 6.2.1 Excess fixative exposure

A high proportion of a strong fixing agent, such as an aldehyde, in a fixative combination may cause the tissue to break down or digest. Tissue that seems “frayed” is indicative of this condition. If the tissue is kept in the first fixative for an extended length of time before being transferred to ethanol storage, similar problems can arise. If the conventional fixative procedure is followed, this issue may be resolved very easily. Additionally, the first fixation period is often brief, lasting between a few hours to a few days. You need to make the most of this time for your specific tissue. Tissue that contains more than 75% ethanol may become stiff, rubber, and difficult to segment.

#### 6.2.2 Insufficient exposure to fixating agent

For long-term preservation, using a low proportion of ethanol (less than 50%) might increase the risk of pathogen infection. For the same reasons, it is not advised to store in water. Using a quick and potent fixative during harvest and before storage may reduce the likelihood of pathogen infection. It takes some time for the fixative to penetrate tissue, whether FAA, ethanol, or both are used. Therefore, if sufficient time is given for fixative penetration, sectioning will be at its best (72–74).

#### 6.2.3 Too young tissue

Compared to older tissues, young plant tissues are significantly more delicate and challenging to handle and segment. Premature growth of vascular cells and, in some cases, the diameter are indicators of young tissue. If immature tissues are needed, the researcher could think about using a microtome to cut them after they have been embedded in paraffin, agar, or another plant tissue (75).

### 6.3 Issues with section adhesion

Sections could not stick to slides well, which could cause staining loss. Reducing the workspace’s humidity and temperature can help to lessen this problem (76) (Table 2).

**Table 2:** Comparison between cutting issues.

Issue	Cause	How to overcome
Angled Cut	Cut not along the longitudinal axis of the root	Provide perpendicular cuts; resurface before continued progress
Cut Too Thin	The section is not fully spherical or has gaps	Tune thickness settings to generate whole, intact pieces
Cut Too Thick	Section borders are thick; many cell layers are visible.	Thin out for cleaner single-layer pieces
Dull Razor Blade	Blade resistance smears or distorts sections	Replace the blade frequently; do not apply excessive pressure or sawing
Half-Cut Sections	Convection in a water drop pulls tissue back to the blade	Tune the blade and water handling; repair/replace the blade if necessary
Excess Fixative Exposure	Excessive aldehyde concentration or over fixation	Follow standard fixation times; fix swiftly in ethanol
Insufficient Fixative Exposure	Insufficient ethanol concentration; under fixation	Use a strong fixative initially; offer sufficient penetration time
Too Young Tissue	Delicate, immature cells are challenging to section.	Fix in paraffin or agar before sectioning; use a microtome.
Poor Slide Adhesion	Sections fail to stick to slides, especially during staining	Dry area around; reduce the humidity and temperature of the workspace

## 7 Applications of microtomy

Microtomes have many applications in histology, pathology, and research applications, and these applications can be summarized as follows:

Conventional Histology where the process involves fixing, drying, clearing, and embedding tissues in molten paraffin, which solidifies into a block when cooled. The connective tissue is then sliced at thicknesses ranging from 2 to 50 μm using a microtome. After the paraffin has been removed, the tissue can then be placed on a slide for the microscope, dyed with the proper aqueous dye or dyes, and inspected under a light microscope (77).

The process of creating frozen sectioning involves freezing water-rich tissues, then cutting them with a freezing microtome or microtome-cryostat. The sections are then stained and seen under a light microscope. This method is used together with medical tests to provide a prompt diagnosis since it is significantly faster than standard histology (5 min vs. 16 h). Because freezing tissue prevents tissue disintegration more quickly than applying an adhesive and does not significantly change or hide its chemical makeup, cryosections are additionally utilized in immunohistochemistry (78).

The electron microscopy technique involves coating tissues in epoxy resin and then cutting extremely thin slices (usually 60 to 100 nanometers) using a microtome fitted with a glass or gem-grade diamond knife. Transmission electron microscopy is used to view the sections after they have been labeled with an aqueous solution of the proper heavy metal salt. This device is frequently referred to as an ultramicrotome. Before thin sectioning, survey sections are also cut using the ultramicrotome’s glass knife or an industrial-grade diamond knife. Before the thin cutting for the TEM, these survey sections, which are typically 0.5 to 1 μm thick, are put on a glass slide and pigmented to identify regions that are important under a light microscope. A diamond knife of gem grade is frequently used for thin cutting for the transmission electron microscope (TEM) (61, 72, 79, 80). Ultramicrotomes, which are positioned within SEM chambers to photograph the block face’s surface and then remove it using the microtome to reveal the next layer for imaging, are becoming more common as a complement to conventional transmission electron microscope (TEM) procedures. Sequential block-face electron microscope (SBFSEM) is the name of this method (81).

Horticultural Microtomy Method (HMM): A sledge microtome is needed for hard materials like leather, bone, and wood. Because of their larger scissors, these microtomes are unable to cut as thinly as a standard microtome machine (82).

For the infrared beam to pass through the specimen being examined, thin polymer slices are required. Using a microtome to cut materials to a thickness of 20 to 100 μm is the standard procedure. Enables more thorough examination of considerably smaller regions in a thin slice where specimen inspection may be done using Fourier transform infrared (FTIR) microscopy (83).

## 8 Future trends in microtome technology

Microtome innovation is a constantly changing subject, with new developments improving both functionality and ease of use. Microtomy’s future is being shaped by the emergence of digital pathology. Real-time tissue specimen examination is made possible by the integration of microtomes with optical technology. Pathologists may compare histology results with digital data thanks to this synergy, which increases the accuracy of diagnosis. Furthermore, more adaptable microtome systems catered to certain research requirements could potentially result from future developments. Dynamically changing the slicing settings might improve research adaptability and make tailored medical strategies possible. Manufacturers are looking at eco-friendly microtome choices as medical research prioritizes sustainability. Disposable blades and energy-efficient layouts that reduce the carbon impact of laboratory operations are examples of innovations (84). Microtome technology appears to have a bright future as research into more automation and artificial intelligence (AI) integration continues. New technologies seek to improve tissue sectioning speed and accuracy, opening the door for breakthroughs that might completely alter research and diagnostic procedures. The relevance of smart microtomes in medical science will only be strengthened by their capacity to adapt to different specimen kinds and cutting needs (85–88, 92).

### 8.1 Automation in sectioning

An innovative technique for handling specimens from anatomical pathology is tissue-sectioning automation. An automated method has the advantages of consistent thickness, consistent orientation, and fewer tissue-sectioning artifacts as compared to traditional hand sectioning. In this brief paper, the layout of an automated tissue-sectioning system is shown, and the sectioned specimens are compared to those that were sectioned manually by a skilled histology professional. The automated system demonstrated satisfactory performance with well-preserved morphological and tissue antigenicity, and it was safe and easy to use. It is anticipated that the turnaround period will be shortened soon (93). Additionally, the combination of microtomes with cutting-edge imaging technologies is opening the door to more precise tissue examination and better understanding of cellular architecture (22, 89).

## 9 Challenges in microtomy

Several factors impact the microtome and the quality of sections and could be summarized as follows (59, 90, 91) (see Table 3).

**Table 3:** Factors that affect the microtome and the quality of sections.

Factor	Description
A. Steady worktop	The bench needs to be strong enough to support the entire weight of the floating bath and microtome, in addition to the microtome’s motion when sectioning. Benches need to be fastened to the wall or floor to prevent vibration-caused artifacts like chatter or bowed regions.
B. Well-maintained microtome	The microtome should be given top attention when it pertains to upkeep and servicing, as even the mildest mechanical malfunction can compromise section quality.
C. Secure parts	All components (blade holder, clamps, etc.) of the microtome should be fastened down to prevent defects by vibration, like washboarding.
D. Correct blade tilt	Adjust clearance angle (3–8°), and usually not required to be adjusted frequently. It depends on the type of blade being used.
E. Sharp blade	Discard the blade with a new one after cutting through blocks or when there are signs of tearing/streaking. Hairy/hardened tissue will drastically shorten a blade’s lifespan.
F. Uniform cutting speed	Maintain a consistent speed to avoid thick/thin sections or washboarding. Tissue sections will differ in thickness and might not form a whole ribbon if a comparable speed of cutting is not applied to all the sections in a single ribbon.
G. Satisfactory water bath	Use fresh, distilled water, 5–10°C below the paraffin melting point.
H. Proper tissue processing	Fixation, processing, and embedding must be done correctly; ill-prepared blocks inhibit quality sectioning. The histopathologists cannot cut an entire and representative segment for diagnosis if the tissue is not implanted totally flat or oriented correctly.
I. Cold, wet blocks	Chill blocks in ice water but never freeze. Blocks should not be kept in the fridge since the tissue and paraffin may split. Tissue slices will acquire artifacts from freezing blocks. Before sectioning, the viewable block surface of blocks with calcium deposits should be decalcified.
J. Controlled environment	Precautions should be taken to avoid drafts and extremely high or low temperatures, or humidity. Even breathing or air currents can blow ribbons away or ruin their shape.
K. Skilled microtomist	Accuracy and patience are required to generate high-quality sections on a predictable basis.

## 10 Conclusion

Acquiring proficiency in sectioning using the slide microtome technique is crucial for biomedical research and histology. We may improve our specimen preparation procedures and further medical knowledge by being aware of the many kinds of microtomes, their principles of operation, and best practices. Professionals in the sector will need to remain up to date on the newest methods and trends as technology develops further.

### 10.1 Commonly related queries

#### 10.1.1 What is the optimal histological slice thickness?

Histological portions should normally have a thickness of 3 to 10 micrometers, according to the analytical needs.

#### 10.1.2 How frequently should microtomes be adjusted?

Regular microtome calibration is advised, preferably following extensive use or when switching among specimen kinds.

#### 10.1.3 Which embedding media are appropriate for sectioning microtomes?

Paraffin wax, resin, and cryo-media for sections that are frozen are examples of common embedding media. The kind of tissue and the intended analysis determine which option is best.

#### 10.1.4 What are some ways to stop sections from curling while being sectioned?

Curling may be avoided by utilizing well-prepared specimens and keeping the workstation at a constant temperature and humidity level.

#### 10.1.5 When utilizing a microtome, are there any precautions one needs to take into account?

Yes, to prevent exposure to harmful chemicals during specimen preparation, always wear the proper personal protection equipment (PPE), such as gloves and goggles, and make sure the microtome is utilized in a well-ventilated location.
